# Unique Clindamycin-Resistant *Clostridioides* *difficile* Strain Related to Fluoroquinolone-Resistant Epidemic BI/RT027 Strain

**DOI:** 10.3201/eid2602.181965

**Published:** 2020-02

**Authors:** Andrew M. Skinner, Laurica Petrella, Farida Siddiqui, Susan P. Sambol, Christopher A. Gulvik, Dale N. Gerding, Curtis J. Donskey, Stuart Johnson

**Affiliations:** Edward Hines, Jr. VA Hospital, Hines, Illinois, USA (A.M. Skinner, L. Petrella, F. Siddiqui, D.N. Gerding, S. Johnson);; Loyola University Medical Center, Maywood, Illinois, USA (A.M. Skinner, S. Johnson);; Centers for Disease Control and Prevention, Atlanta, Georgia, USA (C.A. Gulvik);; Louis Stokes VA Hospital, Cleveland, Ohio, USA (C.J. Donskey);; Case Western Reserve University, Cleveland (C.J. Donskey)

**Keywords:** Clostridium difficile, restriction endonuclease analysis, REA, Clostridioides difficile, outbreak, BI, RT027, bacteria, antimicrobial resistance, United States

## Abstract

During a surveillance study of patients in a long-term care facility and the affiliated acute care hospital in the United States, we identified a *Clostridioides difficile* strain related to the epidemic PCR ribotype (RT) 027 strain associated with hospital outbreaks of severe disease. Fifteen patients were infected with this strain, characterized as restriction endonuclease analysis group DQ and RT591. Like RT027, DQ/RT591 contained genes for toxin B and binary toxin CDT and a *tcdC* gene of identical sequence. Whole-genome sequencing and multilocus sequence typing showed that DQ/RT591 is a member of the same multilocus sequence typing clade 2 as RT027 but in a separate cluster. DQ/RT591 produced a similar cytopathic effect as RT027 but showed delayed toxin production in vitro. DQ/RT591 was susceptible to moxifloxacin but highly resistant to clindamycin. Continued surveillance is warranted for this clindamycin-resistant strain that is related to the fluoroquinolone-resistant epidemic RT027 strain.

As the leading cause of healthcare-associated infectious diarrhea and colitis, *Clostridioides* (formerly *Clostridium*) *difficile* continues to affect patients in hospitals and extended-care facilities in the United States (*1*–*3*). Among the numerous *C. difficile* strains, none have been more important to the healthcare community at large than the strain characterized as toxinotype III, restriction endonuclease analysis (REA) group BI, PCR ribotype (RT) 027, and sequence type (ST) 1, also known as pulsed-field gel electrophoresis type NAP1 (*4*,*5*). Because the BI/RT027 strain has been associated with numerous healthcare facility outbreaks and an increasing number of illnesses and deaths, it has been called hypervirulent (*6*). Factors that potentially contribute to increased virulence for this strain include increased sporulation, particular DNA gyrase mutations, the ability to survive in hostile environments, and increased toxin production (*7*–*9*). 

The pathogenicity locus of BI/RT027 includes a characteristic 18-bp deletion and a single-base deletion at position 117 of the *tcdC* gene (*4*). These mutations result in a truncated TcdC protein that is rendered nonfunctional, leading to a lack of regulation of the *tcdA* and *tcdB* genes and potentially increased toxin A and B production (*4*). Virulence may be further affected by the binary toxin (CDT), which is coded for by genes located outside the pathogenicity locus, and its proposed role is to increase adherence of the bacterium to the epithelium (*4*,*10*). 

Presumptive detection of the BI/RT027 strain by the Xpert *C. difficile* EPI assay (Cepheid, https://www.cepheid.com) is based on PCR amplification of targets, including the deletion at position 117 within *tcdC* and sequences within *tcdB* and the binary CDT genes (*11*). During the past 10 years, non-RT027 strains have emerged that test positive by this assay because they have the same or similar gene targets (*12*,*13*). Several of these non-RT027 strains also have been associated with increased numbers of illnesses and deaths, suggesting that the gene targets for this assay may be related to increased virulence (*12*–*14*).

In 2012, during a surveillance study at 2 US Veteran Affairs (VA) long-term care facilities (LTCFs) and their affiliated acute care facilities, we detected a clonal *C. difficile* outbreak at 1 of the LTCFs and the affiliated acute care facility (*15*). The organism was initially identified as the epidemic strain BI/RT027 after it tested positive by Xpert *C. difficile* Epi assay (*15*). However, further analysis identified this strain as REA group DQ and RT591. We report the microbiological and molecular characterization of this strain and the clinical findings of the infected patients.

## Methods

During February 2012–August 2012, we obtained swab specimens from the perirectal area of asymptomatic LTCF patients at 2 VA facilities in Cleveland, Ohio, and Chicago, Illinois, USA, as part of a validation study comparing PCR using the Xpert *C. difficile* Epi assay and culture (*15*). In addition, fecal specimens from symptomatic patients with *C. difficile* infection (CDI) were cultured for *C. difficile* as part of a larger surveillance study of *C. difficile* in each LTCF and the associated acute care facility at these 2 sites (*16*). Specimens obtained from the perirectal area using BD BBL CultureSwabs (Becton Dickinson, https://www.bd.com) and submitted fecal specimens from symptomatic patients were cultured anaerobically on selective media as previously described (*15*). We reviewed medical records to obtain information about demographics, medical conditions, medications, CDI treatment, and outcomes. The severity of CDI and determination of initial versus recurrent cases were classified in accordance with the Infectious Diseases Society of America and Society for Healthcare Epidemiology of America *C. difficile* infection guidelines (*17*). The institutional review board of each hospital approved the study protocol.

### REA

Recovered *C. difficile* isolates were first subjected to typing by REA. Using the methods provided by Clabots et al. (*18*), total cellular DNA was purified and subjected to *Hin*dIII restriction enzyme digestion and electrophoresis. The resulting banding patterns were then compared with a known database. All isolates representing a newly recognized REA type that corresponded to a presumptive BI/RT027 identified by the Xpert *C. difficile* Epi assay were subjected to PCR ribotyping, whole-genome sequencing (WGS), multilocus sequence typing (MLST), and PCR amplification of *cdtA*, *cdtB*, *tcdA*, and *tcdB* and an 18-bp deletion in *tcdC.* Sequencing of *tcdC* (1 isolate*)*, toxinotyping (1 isolate), toxin production in vitro (1 isolate per REA strain group), and antimicrobial susceptibility testing (6 isolates) were performed on selected isolates.

### PCR Ribotyping

We characterized recovered *C. difficile* isolates using high-resolution capillary gel electrophoresis–based PCR ribotyping. We analyzed these isolates against a library of standard profiles, as described previously in the internationally validated consensus protocol from Fawley et al. (*19*).

### Gene Analysis of Binary Toxin CDT, Toxins A and B, and the Negative Toxin Regulator

We conducted PCR amplification of *cdtA*, *cdtB*, *tcdA*, and *tcdB* and an 18-bp deletion in *tcdC* on all isolates identified as BI/RT027 and DQ/RT591 as previously described (*20*). In addition, by using primers previously described by Rupnik et al. (*21*), we amplified *cdtB* and *tcdB* by PCR to confirm the presence of CDT and toxin B on a representative DQ/RT591 isolate followed by amplification and sequencing of the *tcdC* gene. A full-length *tcdC* PCR was performed using the following primers to produce a 910-bp product: forward primer ACTGTTTATTTGCAATTATAAAAACATCT; reverse primer TTACTTTATTTTGTAAAATTATGCTTAGGG. PCR amplicons were gel purified, sequenced, and compared with BI and strain 630.

### Toxinotyping

We conducted toxinotyping on a representative DQ/RT591 isolate by performing restriction fragment-length polymorphism PCR of the B1 and A3 fragment. We assessed for variation in the first 3-kbp of *tcdB* and a repetitive 3-kbp fragment in *tcdA* (*21*).

### WGS and MLST

We conducted WGS using a Nextera kit (Illumina, https://www.illumina.com) to prepare genomic DNA libraries and sequenced the RT591 organism using an Illumina MiSeq producing 2×250-bp read sets in accordance with the manufacturer’s protocols. Reads were filtered with SolexaQA++ 3.1 by dynamic trimming bases lower than Phred 30 and discarding reads <50-bp long. (*22*) We then assembled high-fidelity filtered reads into contigs at least 500 bp long with SPAdes 3.6.2 (*22*,*23*). Isolates that met the following 5 molecular testing criteria (positive for *cdtA*, *cdtB*, *tcdA*, and *tcdB* and an 18-bp deletion in *tcdC*) were then analyzed by genomewide average nucleotide identity (ANI). We computed pairwise ANI values between genomes from NUCmer v3.1 alignments aided by PyANI version 0.1 (*24*). Pairwise identity values were sorted into a 2-dimensional matrix with pandas version 0.15.2 in Python, and a heatmap of identity values with hierarchical clustering linkages was visualized with gplots version 2.16.0 in R (*25*). DQ/RT591 genomes also had single-nucleotide polymorphisms (SNPs) identified with Parsnp version 1.3.

We retrieved all STs on PubMLST’s webserver (https://pubmlst.org/cdifficile) (351 as of June 28, 2016) to compare distances among STs. The database of profiles was based on the first scheme created for *C. difficile*, which uses the loci of *adk*, *atpA*, *dxr*, *glyA*, *recA*, *sodA*, and *tpi*. Of the 3,501-bp length for each ST, 524 positions were found to have a nucleotide variant among the 351 STs. The variable sites were given to RAxML version 8.1.17 with the generalized time reversible substitution model, and the tree was visualized in Figtree version 1.4.2 (*26*).

### In Vitro Toxin Production and Antimicrobial Susceptibility Testing

We determined toxin production on *C. difficile* isolate supernatants of representative isolates of 5 different REA group strains after 24, 48, and 72 hours of growth in brain heart infusion broth media (*27*). Toxin concentrations were determined by enzyme immunoassay (*C. difficile* toxA/B II EIA; TechLab, https://www.techlab.com) and interpolation from a standard curve using a toxin A standard of known concentration. Assays were performed in triplicate on a representative DQ isolate and compared with supernatants from toxigenic strains BI (RT027), J (RT001), AF (RT244), and a nontoxigenic strain, REA group T. A qualitative cytotoxin analysis was performed on the representative isolate supernatants using human fibroblast cells (Bartels cytotoxicity assay; Trinity Biotech, https://trinitylifesciences.com). We determined antimicrobial susceptibilities by Etest (bioMérieux, https://www.biomerieux-usa.com) for moxifloxacin, ceftriaxone, azithromycin, and clindamycin on taurocholate fructose agar plates (*28*,*29*). We confirmed the susceptibility results for moxifloxacin and clindamycin by testing 5 additional unique strains of BI and DQ recovered from patients at the VA site where the DQ outbreak occurred. We used Clinical and Laboratory Standards Institute guidelines for resistance cutoff values (*30*,*31*).

### Statistical Analysis

We compared characteristics and outcomes of patients colonized or infected with BI/NAP1/027 and other strain types with those of patients colonized or infected with DQ/591 strains. Student *t*-test was used for normally distributed data and Fisher exact test for categorical data. We analyzed data using SPSS Statistics 10.0 (IBM, https://www.ibm.com).

## Results

REA group BI strains were the most common strains recovered from both VA sites and accounted for 33 (40%) of the 83 isolates at the Cleveland site. In addition, 16 (19%) of the isolates from 15 Cleveland patients were identified as REA strain DQ, even though the corresponding Xpert *C. difficile* Epi assay results indicated the presence of the NAP1 strain (i.e., REA group BI). No DQ strains were found at the Chicago site.

We compared baseline characteristics and outcomes of the 15 patients with fecal cultures positive for the DQ strain with those of the 22 patients with BI/NAP1/027 strains and 27 with other strain types (Table). Ten (67%) of the 15 patients with the DQ strain were LTCF residents, 4 (27%) were on the spinal cord injury unit, and 1 was hospitalized on a medical ward. Of the 7 patients with CDI caused by DQ strains, 3 (43%) met criteria for severe CDI, but none had fulminant CDI. All 7 CDI cases were healthcare associated; 3 of these patients had onset in the hospital, and 4 had onset in the LTCF. In all patients with CDI caused by DQ strains, diarrhea resolved with therapy, but CDI recurred in 3. Patients colonized or infected with DQ strains were significantly more likely than those with BI/NAP1/027 or other strain types to be LTCF residents and to have received antimicrobial drugs during the past 90 days. Patients with other strain types were significantly less likely than patients with DQ strains to have a recent intensive care unit admission, to have healthcare-associated CDI, or to die within 6 months after the CDI diagnosis.

**Table:** Comparison of baseline characteristics and outcomes of patients colonized or infected with *Clostridioides difficile* DQ/591, BI/NAP1/027, and other strain types in study of C. *difficile* at 2 US Veteran Affairs long-term care facilities and their affiliated acute care facilities*

Characteristic	Strain type		
	DQ/591, n = 15	BI/027, n = 22	Other, n = 27
Age, y (range)	67.9 (49–85)	68.9 (57–89)	67.4 (48–91)
Sex			
M	15 (100)	22 (100)	26 (96)
F	0	0	1 (4)
Residence			
Long-term care facility	10 (67)	7 (32)†	8 (30)†
Spinal cord injury unit	4 (27)	8 (36)	7 (26)
Drugs received			
Proton pump inhibitor	12 (80)	11 (50)	19 (70)
Antimicrobial drug treatment in past 90 d	15 (100)	14 (64)†	15 (56)†
Fluoroquinolone in past 90 d	5 (33)	6 (27)	6 (22)
Clindamycin in past 90 d	1 (7)	0	0
Azithromycin in past 90 d	1 (7)	0	0
Intensive care unit admission in past 30 d	4 (27)	4 (18)	0†
Medical conditions			
Chronic lung disease	6 (40)	4 (18)	5 (19)
Cancer	5 (33)	5 (23)	5 (19)
Major surgery in past 90 d	3 (20)	1 (4.5)	5 (19)
End-stage renal disease	3 (20)	4 (18)	4 (15)
Disease classification, no. (%)			
*C. difficile* infection	7 (47)	13 (59)	16 (59)
Severe	3 (43)	3 (23)	1 (6)
Fulminant	0	0	0
Recurrent	3 (43)	2 (15)	4 (25)
Healthcare-associated	7 (100)	11 (85)	8 (50)†
Asymptomatic carrier	8 (53)	9 (41)	11 (41)
Died within 6 mo after CDI diagnosis	4 (27)	6 (27)	1 (4)†

*Values for BI/027 and for other strain types were compared with values for DQ/591 strains. †p<0.05.

**Figure 1:**
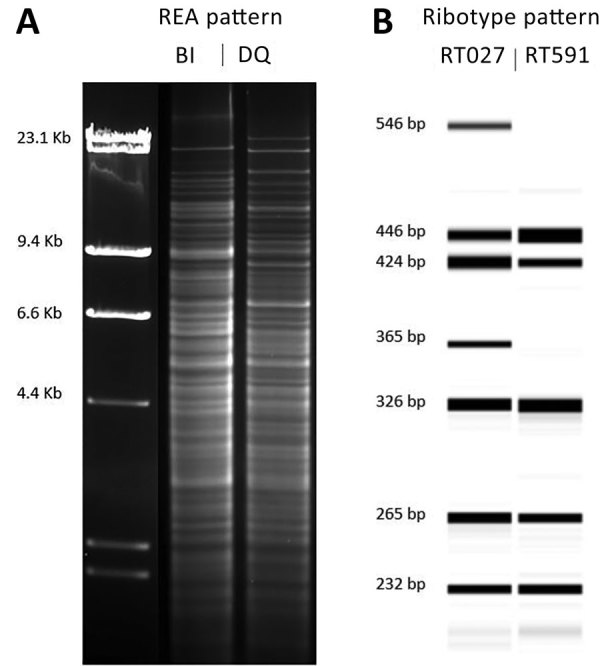
Comparison of the molecular characteristics of *Clostridioides difficile* strain DQ/591 and the epidemic BI/027 strain in study of C. *difficile* at 2 US Veteran Affairs long-term care facilities and their affiliated acute care facilities. The *Hin*dIII REA (A) and PCR ribotype (B) banding patterns were distinct between REA strain DQ/RT591 and REA strain BI/RT027. Molecular weight markers (in kb) are shown adjacent to the REA gel pattern. An internal spiked LIZ 1200 standard was used for fragment length calibrations (in bp) of the PCR ribotype gel patterns. REA, restriction endonuclease analysis.

The *Hin*dIII REA banding patterns differed between the DQ and BI strains (Figure 1, panel A). The PCR ribotype patterns were more similar, except for the RT591 pattern (DQ), which was missing 2 major bands present in RT027 pattern (BI) (Figure 1, panel B).

PCR amplification of *tcdB* and *cdtB* in a representative DQ isolate indicated the presence of genes for toxin B and binary toxin CDT, consistent with the profile of the epidemic BI/RT027 strain (data not shown). In addition, amplification and sequencing of the *tcdC* gene showed complete alignment with *tcdC* from a reference BI strain, both of which contained an 18-bp deletion from positions 316 to 333 and a single base deletion at position 117 that resulted in a stop codon at position 196, unlike the reference *C. difficile* strain, 630 (data not shown) (*32*). The similarities to the BI/RT027 strain were further validated because toxinotyping indicated the DQ/591 strain was toxinotype III (data not shown).

**Figure 2:**
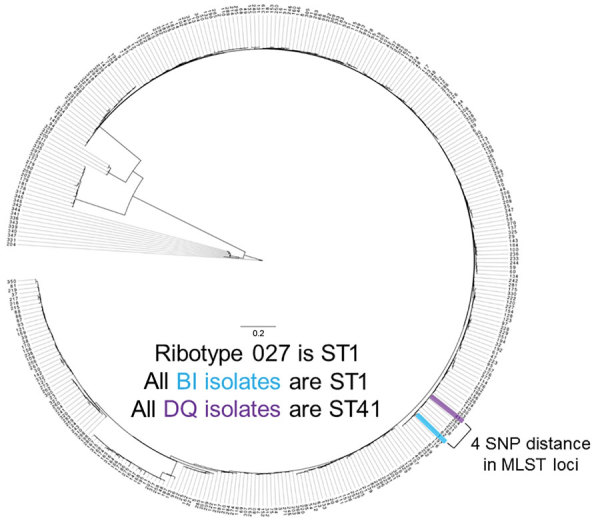
MLST loci map for *Clostridioides difficile* strains DQ/RT591 and BI/RT027 in study of *C.* *difficile* at 2 US Veteran Affairs long-term care facilities and their affiliated acute care facilities. The 2 strains are 4 SNPs apart. Scale bar indicates nucleotide substitutions per variable site of loci. MLST, multilocus sequence typing; SNP, single-nucleotide polymorphism; ST, sequence type.

WGS and MLST results showed that, although the strains are closely related and reside within the same clade (MLST clade 2), they form a separate cluster (Figure 2). ANI showed a clear separation of the BI/027 and DQ/591 isolates at the whole genome level (Appendix Figure 1). By MLST analysis, the DQ/RT591 isolates were ST41, whereas BI/RT027 were ST1. Although the STs are closely related, there is a 4 SNP separation in the MLST loci.

**Figure 3:**
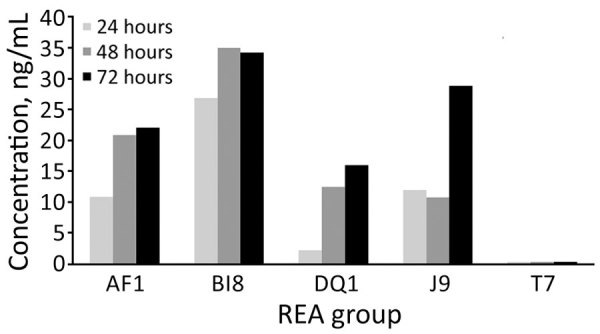
Quantitative in vitro total toxin production in study of *C.* *difficile* at 2 US Veteran Affairs long-term care facilities and their affiliated acute care facilities. Results at 24, 48, and 72 hours of incubation are shown for REA strains AF (ribotype 244), BI (ribotype 027), DQ (ribotype 591), J (ribotype 001), and T (a nontoxigenic *Clostridioides difficile* strain). REA, restriction endonuclease analysis.

Supernatant from the DQ/RT591 strain produced typical cellular rounding on fibroblasts similar to BI/RT027, whereas the AF/RT244 strain produced a different phenotype, clumping and rounding of cells (Appendix Figure 2). Strain AF/RT244 is also related to RT027 and was responsible for an outbreak of severe disease in Australia (*12*). DQ/RT591 showed minimal toxin production in vitro at 24 hours; by 48 hours, toxin levels were similar to those of strain J/RT001 but still less than levels produced by BI/RT027 (Figure 3).

The DQ/RT591 strain was highly resistant to clindamycin (MIC >256 μg/mL) and azithromycin (MIC >256 μg/mL) but was susceptible to moxifloxacin (MIC range 1–2 μg/mL) and ceftriaxone (MIC 16 μg/mL). BI/RT027 was resistant to moxifloxacin (MIC >32 μg/mL) and azithromycin (MIC >256 μg/mL) and variably resistant to clindamycin (MIC range 6–32 μg/mL). Furthermore, WGS revealed that all DQ/RT591 isolates contained a variant *ermB* sequence known to confer clindamycin resistance (*33*).

## Discussion

During a surveillance study of *C. difficile* in asymptomatic LTCF patients and symptomatic patients in the affiliated acute care hospitals at 2 VA facilities (*16*), we detected a clonal outbreak of a newly recognized *C. difficile* strain at the Cleveland facility. This strain, identified as REA group DQ, ribotype 591, is closely related to the BI/RT027 strain, and a commercial PCR erroneously identified it as the epidemic NAP1 strain (i.e., BI/RT027). The strain was misidentified as NAP1 by Xpert *C. difficile* PCR (Cepheid) because of the similar genetic findings within the *C. difficile* pathogenicity locus, and the presence of the binary toxin CDT gene *cdtB* (*33*). Our findings and previous reports of other non-RT027 strains show that these genetic targets are not specific to NAP1 (*12*,*13*). Even though the outbreak of RT244 in Melbourne, Australia, was misidentified as NAP1 (RT027) by the same commercial PCR, it was associated with severe disease in the infected patients (*12*), suggesting the possibility of shared virulence determinants.

DQ/RT591 and BI/RT027 share several characteristics, including *tcdB*, *cdtB*, the 18-bp and position 117 *tcdC* deletions, and a similar cytotoxic phenotype. Despite these shared genetic and phenotypic characteristics, in vitro toxin production appeared to be delayed and somewhat lower in the DQ/RT591 strain than in BI/RT027. The clinical manifestations of the patients colonized or infected with DQ/RT591 were not unusual and did not differ substantially from those of patients with BI/NAP1/027 and other strain types in the cohort reported here. In nearly half (47%) of the patients, symptoms developed that were consistent with CDI. Although no fulminant CDI cases or deaths directly related to *C. difficile* were recognized, in CDI that developed because of DQ/591, 43% were classified as severe CDI in accordance with Infectious Diseases Society of America and Society for Healthcare Epidemiology of America guidelines. Likewise, no fulminant CDI cases or CDI-related deaths were recognized among the patients infected with BI/RT027 during this same study. However, all-cause mortality was lower for persons with non-DQ/RT591 and non-BI/RT027 infections. All the documented transmission events occurred in the BI/RT027 patients (*34*). Despite the fact that all the CDI cases caused by DQ/RT591 were healthcare associated, WGS did not identify any transmission events between patients because core genome SNP differences were >8 among DQ isolates (Appendix Figure 3) (*34*).

All patients in whom DQ/RT591 was confirmed had received antimicrobial drugs within 90 days before testing, and all patients with a CDI were classified as having a healthcare-associated infection; nearly all cases occurred in the LTCF or spinal cord injury unit. No specific antimicrobial drug was highly associated with infection by this strain. Fluoroquinolones have been associated with CDI outbreaks with the BI/RT027 strain, which is highly resistant to fluoroquinolones in vitro (*35*). Among the DQ/RT591- and BI/RT027-infected patients, receipt of fluoroquinolones or clindamycin was limited. Despite high-level resistance to clindamycin in vitro and the presence of *ermB* in DQ/RT591, only 1 patient infected with this strain had received clindamycin.

Our experience was limited, but DQ/RT591 did not appear to carry the same level of clinical severity that BI/RT027 has exhibited (*6*). This difference in severity might be attributable to delayed toxin production in vivo (Figure 3). Although increased toxin production has been proposed as the reason for increased virulence with the BI/RT027 strain (*4*,*29*), increased toxin production was not demonstrated for the AF/RT244 strain, which nevertheless was associated with an outbreak of increased severity in Australia (*12*). AF/RT244 also shows a different cytopathic effect than BI/027, which suggests the presence of a variant toxin B in AF/RT244 (*4*,*12*). Factors associated with increased virulence associated with these strains are still incompletely defined.

Because REA DQ/RT591 is closely related to BI/027, further monitoring is required to determine whether this strain carries risk for increased illness and death or has the capability of widespread dissemination. Since we completed this work, RT591 was reported as the most prevalent *C. difficile* strain in 3 tertiary hospitals in Colombia. These RT591 isolates were also mostly resistant to clindamycin (85%) (*36*).

Appendix Additional results for clindamycin-resistant *Clostridioides difficile* strain DQ/RT591.
