# Supplementary material for: Unique Clindamycin-Resistant Clostridioides difficile Strain Related to Fluoroquinolone-Resistant Epidemic BI/RT027 Strain
Source: Emerg Infect Dis. 2020 Feb;26(2):247–54. doi: 10.3201/eid2602.181965 (PMC6986856; doi:10.3201/eid2602.181965)
Supplement: Appendix — Additional results for clindamycin-resistant Clostridioides difficile strain DQ/RT591. [file 18-1965-Techapp-s1.pdf]

Article DOI: https://doi.org/10.3201/eid2602.181965

Unique Clindamycin-Resistant *Clostridioides difficile* Strain Related to Fluoroquinolone-Resistant Epidemic BI/RT027 Strain

Appendix

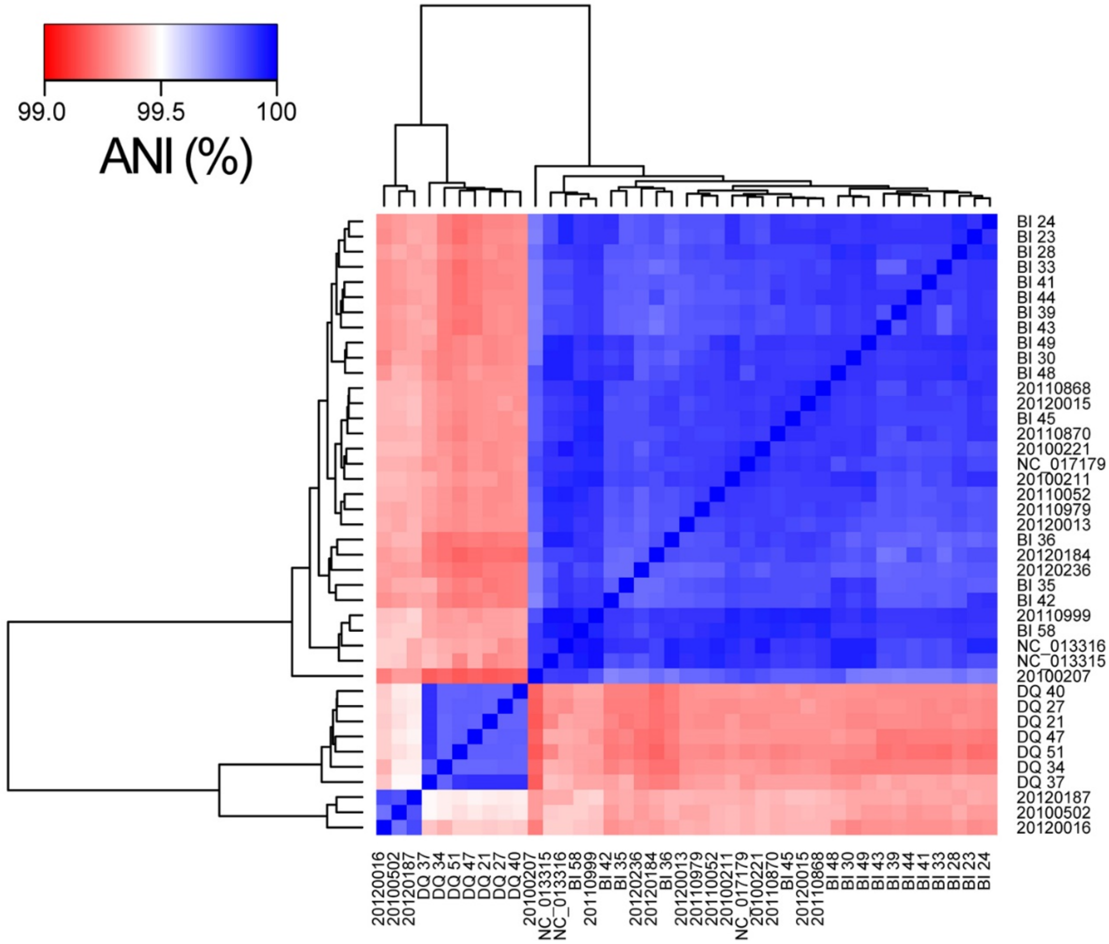

Appendix Figure 1. Pairwise ANI comparisons demonstrate clear partitioning of DQ/RT591 and BI/RT027 isolates at the whole-genome level. Strains with a BI or DQ prefix were recovered from the outbreak (n = 23) and were PCR ribotyped but named according to our REA results, NC_ prefixed strains are public reference genomes retrieved from NCBI GenBank(n = 3), and the remaining numbered strains

(n = 15) are genomes from our NCBI BioProject 296517 that we typed by PCR ribotyping and correspond to RT027, which cluster with the BI strains or correspond to RT591, which cluster with the DQ strains. ANI, average nucleotide identity.

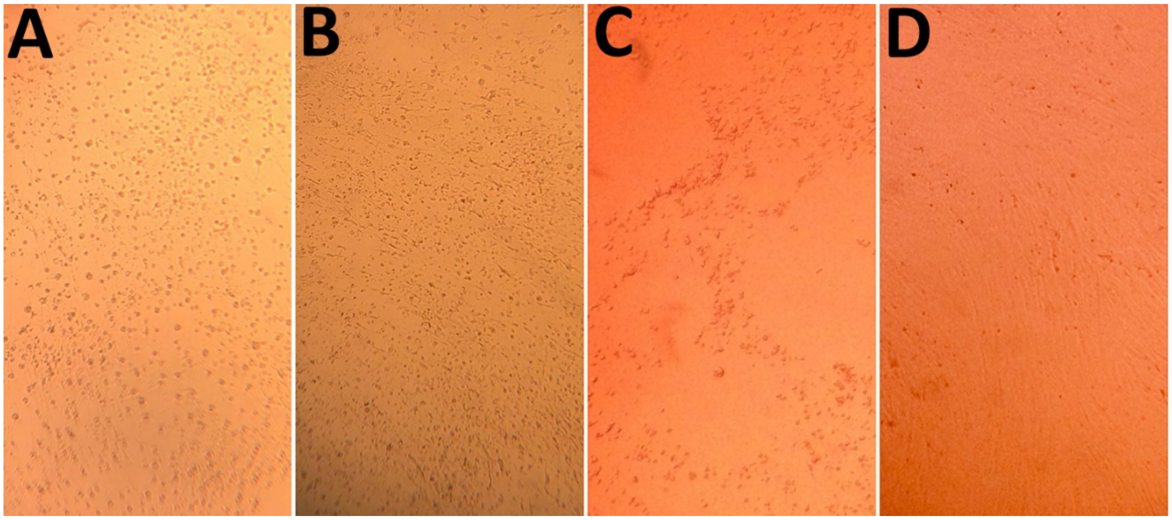

Appendix Figure 2. Cytopathic effect of supernatants from toxigenic *Clostridioides difficile* strains DQ/RT591(A), BI/RT027(B), and AF/RT244 (C) on human fibroblast cells compared with the noncytopathic effect of toxin A/B negative *C. difficile* restriction endonuclease analysis (REA) group T strain (D). Note the atypical cytopathic effect of AF/RT244 with the rounding and clumping of the fibroblasts. The cytopathic effects of DQ/RT591 and BI/RT027, both of which are toxinotype III strains, are identical to the cytopathic effect of toxinotype 0 strains (data not shown). The atypical cytopathic effect seen with AF/RT244 (toxinotype IX [*1*]) also has been seen in toxinotype VIII strains that produce a variant toxin B (*2*).

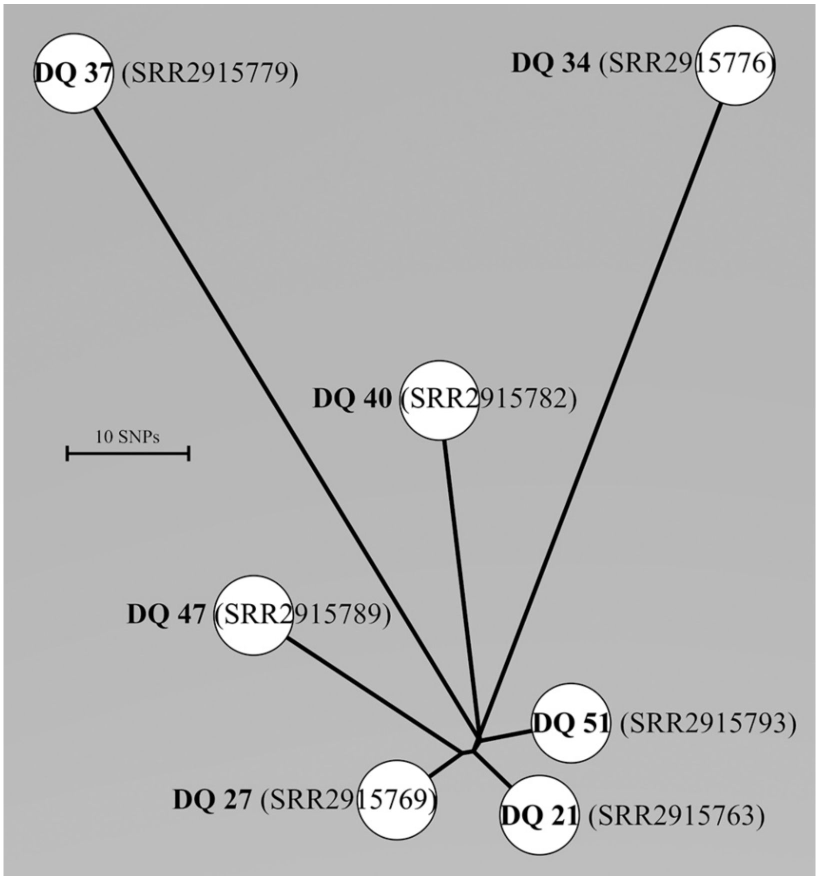

Appendix Figure 3. Minimum-spanning tree of core genomic single-nucleotide polymorphism (SNP) sites for 7 DQ/RT591 isolates. Sample identifiers are in bold, and the corresponding NCBI GenBank accession numbers are in parentheses. Scale bar indicates 10 SNPs. The mean SNP distance between isolates was 38 (range 8–79).

References

1. Rupnik M, Janezic S. An update on *Clostridium difficile* toxinotyping. J Clin Microbiol. 2016;54:13–8. PubMed https://dx.doi.org/10.1128/JCM.02083-15

2. von Eichel-Streiber C, Zec-Pirnat I, Grabnar M, Rupnik M. A nonsense mutation abrogates production of a functional enterotoxin A in *Clostridium difficile* toxinotype VIII strains of serogroups F and X. FEMS Microbiol Lett. 1999;178:163–8. PubMed https://dx.doi.org/10.1016/S0378-1097(99)00327-4
